# 

**Published:** 1906-10

**Authors:** 


### CONTENTS.

ORIGINAL COMMUNICATIONS—

REPORT OF AN INTERESTING CASE. By Dunbar Roy, M.D., Atlanta, Ga. .......... 433
PROGNOSIS IN PULMONARY TUBERCULOSIS. By Mary Lapham, M.D., Highlands, N. Y. .......... 441

CORRESPONDENCE—

BRITISH MEDICAL ASSOCIATION MEETING .......... 451

EDITORIAL NOTES AND COMMENTS—

CASTRATION INSTEAD OF LYNCHING. .......... 456

NEWS AND NOTES .......... 459

BOOK REVIEWS—

A TEXT BOOK OF HUMAN PHYSIOLOGY. (Tigerstedt.) .......... 463

CONTENTS CONTINUED .......... X

